# Effect of Anesthesia Type on Clinically Significant Prostate Cancer Detection in Transperineal MRI-Targeted Biopsy

**DOI:** 10.3390/cancers18172820

**Published:** 2026-09-01

**Authors:** Shijie Jin, Dake Pan, Yuzhi Zuo, Yi Zhou, Zhien Zhou, Weigang Yan

**Affiliations:** Department of Urology, Peking Union Medical College Hospital, Beijing 100730, China

**Keywords:** prostate biopsy, transperineal, local anesthesia, general anesthesia, cancer detection

## Abstract

Background/Objectives: The transperineal approach is the recommended standard for prostate biopsy. We aimed to determine whether anesthesia type—local anesthesia (LA) versus general anesthesia (GA)—independently influences clinically significant prostate cancer (csPCa) detection in transperineal magnetic resonance imaging (MRI)-targeted biopsy and to evaluate its impact on biopsy–prostatectomy pathological concordance. Methods: This single-center retrospective study included biopsy-naive men undergoing cognitive-fusion transperineal prostate biopsy under LA or GA. Propensity score matching (1:1) was performed on age, prostate-specific antigen, PI-RADS score, and prostate volume. The primary outcome was csPCa detection (ISUP Grade Group ≥ 2) on combined systematic and targeted biopsy. Secondary outcomes included overall and high-grade cancer detection and biopsy–prostatectomy pathological concordance. Results: After matching, 220 pairs (440 patients) were analyzed. csPCa detection on combined biopsy did not differ between LA and GA (40.5% vs. 37.7%, *p* = 0.625; absolute risk difference −2.7 percentage points, 95% CI −11.8 to 6.4; relative risk 0.93, 95% CI 0.74–1.18), nor did overall (54.5% vs. 55.0%, *p* = 1.000) or high-grade cancer detection (21.4% vs. 24.1%, *p* = 0.569). Similarly, no significant differences were observed for systematic biopsy alone (csPCa: 36.4% vs. 34.5%, *p* = 0.765) or targeted biopsy alone (csPCa: 35.9% vs. 35.0%, *p* = 0.921). Subgroup and multivariable analyses showed that anesthesia type was not an independent predictor of csPCa (adjusted odds ratio 0.887, 95% CI 0.553–1.423, *p* = 0.620). Biopsy–prostatectomy pathological concordance did not differ significantly between groups in the 121 prostatectomy patients. Conclusions: Anesthesia type was not significantly associated with csPCa detection in transperineal cognitive-fusion biopsy, and pathological concordance was similar between LA and GA. These findings support the feasibility of LA transperineal biopsy, although the confidence intervals do not establish formal equivalence between the two approaches.

## Simple Summary

Prostate biopsy is essential for diagnosing prostate cancer, and the transperineal approach is increasingly preferred because it substantially reduces the risk of infectious complications. This procedure can be performed under either local anesthesia or general anesthesia. It remains unclear whether the type of anesthesia affects the ability to detect clinically important prostate cancers. In this study, we compared cancer detection rates between 220 patients who underwent transperineal biopsy under local anesthesia and 220 matched patients who received general anesthesia. We found no statistically significant difference in the detection of clinically significant prostate cancer between the two groups, and the accuracy of biopsy grading compared with final surgical pathology was also similar. These findings suggest that local anesthesia transperineal biopsy achieves cancer detection outcomes that do not differ significantly from those achieved under general anesthesia, supporting the continued expansion of the use of LA transperineal biopsy without an observed reduction in diagnostic yield.

## 1. Introduction

Prostate cancer is the second most common cancer in men worldwide and a leading cause of cancer-related mortality [1]. Prostate biopsy remains the cornerstone of prostate cancer diagnosis. Over the past decade, clinical practice has undergone a fundamental paradigm shift from the transrectal ultrasound-guided (TRUS) route toward the transperineal (TP) approach, driven principally by mounting evidence that TP biopsy substantially reduces the risk of post-biopsy sepsis [2] and may improve sampling of anterior and apical tumors [3,4]. The European Association of Urology (EAU) now strongly recommends the TP approach as the preferred initial biopsy strategy [5], and several national health systems have committed to transitioning away from TRUS biopsy [4,6].

The TP approach may be performed under either local anesthesia (LA) or general anesthesia (GA). While GA eliminates the need for patient cooperation and avoids the discomfort associated with perineal skin puncture and pelvic floor manipulation, optimized LA protocols incorporating perineal infiltration, periprostatic nerve block (PPNB), and pudendal nerve block have enabled the development of office-based LA-TP biopsy [7,8]. LA-TP biopsy has been shown to be a safe and well-tolerated procedure, with over 95% of procedures completed successfully and pooled csPCa detection rates of approximately 48% [7,8]. Proponents of GA argue that the controlled operative environment permits more deliberate targeting of MRI-visible lesions, particularly those in the anterior and apical zones where needle trajectory adjustments may require greater patient cooperation [7]. Furthermore, periprostatic nerve block may provoke vasovagal episodes in approximately 2–5% of patients, which, although usually self-limiting, can interrupt the procedure and compromise operator workflow [9]. Survey data indicate that over two-thirds of urologists who do not perform LA-TP biopsy cite concerns regarding procedural duration and patient tolerability as primary deterrents [10].

To date, however, only a limited number of studies have directly compared LA and GA within a uniform TP biopsy framework. Hogan et al. reported comparable overall cancer detection rates (55% vs. 55%) and csPCa detection (35% vs. 22.5%, *p* = 0.217) between LA and GA transperineal biopsy in a prospective pilot study of 80 patients [7]. Kim et al. retrospectively compared propofol sedation with intrarectal lidocaine gel in 2119 TRUS biopsies and found a significant difference in overall cancer detection (34.0% vs. 29.2%, *p* = 0.024 after adjustment) [11]. Lv et al., in the only randomized controlled trial directly comparing LA-TP versus GA-TP biopsy to date (n = 216), reported no significant difference in cancer detection between the two approaches (41.7% vs. 39.8%, *p* = 0.782) [12]. A more recent multicenter retrospective analysis by Vasconcelos Ordones et al. compared three MRI-targeted TP biopsy strategies across 1272 patients and unexpectedly observed higher csPCa detection with in-office LA cognitive-fusion biopsy than with GA software-assisted fusion biopsy (51% vs. 37%, *p* < 0.001), although operator experience was a significant confounder [13]. A 2024 Health Technology Assessment commissioned by NICE confirmed the absence of further comparative trials beyond the Lv et al. RCT [12], concluding that evidence for this specific comparison remains sparse [14]. Most recently, a large single-center series presented at the 2025 AUA Annual Meeting reported comparable csPCa detection between LA (43%) and GA (45%, *p* = 0.3) in 1021 men, noting that higher intra-procedural pain during LA was associated with lower csPCa detection on interaction testing [15].

However, several questions remain unanswered. First, prior studies have reported cancer detection only for combined biopsy, without examining systematic and targeted biopsy separately—yet these two sampling strategies may be differentially influenced by anesthesia-related factors. Second, no study has assessed whether anesthesia type affects the pathological concordance between biopsy and radical prostatectomy. Third, the largest comparative series [13,15] differed in biopsy technique and operator experience between groups. To address these gaps, we conducted a retrospective cohort study of biopsy-naive men undergoing uniform cognitive-fusion TP biopsy. Using propensity score matching, we examined whether anesthesia type independently influences csPCa detection in combined, systematic, and targeted biopsy separately, and evaluated its impact on biopsy–prostatectomy pathological concordance.

## 2. Materials and Methods

### 2.1. Study Design and Population

This was a single-center, retrospective cohort study, conducted in accordance with the STROBE guidelines [16], of consecutive patients who underwent magnetic resonance imaging (MRI)-targeted transperineal prostate biopsy at Peking Union Medical College Hospital between September 2024 and June 2026. The study was approved by the Institutional Review Board of Peking Union Medical College Hospital, and the requirement for informed consent was waived given the retrospective nature of the analysis.

Patients were eligible if they met all of the following criteria: (1) male sex, age < 80 years, with clinical suspicion of prostate cancer based on elevated prostate-specific antigen (PSA), abnormal digital rectal examination (DRE), or both; (2) pre-biopsy multiparametric MRI (mpMRI) demonstrating at least one PI-RADS version 2.1 category ≥3 lesion [17]; and (3) biopsy-naive status (first-time prostate biopsy). Exclusion criteria included PSA ≥ 20 ng/mL, prior prostate biopsy of any kind, and incomplete imaging or pathological data.

### 2.2. Biopsy Procedure

Pre-biopsy mpMRI was performed on a 1.5 T and a 3.0 T MRI scanner (Siemens Healthineers, Erlangen, Germany; GE Healthcare, Chicago, IL, USA; Philips Healthcare, Best, The Netherlands) and assessed by two radiologists with more than 5 years of experience according to PI-RADS v2.1. Prostate volume was calculated using the ellipsoid formula, and PSA density (PSAD) was calculated as serum PSA divided by prostate volume. All biopsies were performed via the transperineal approach using 18-gauge, 18 mm depth Bard biopsy guns (C.R. Bard, Covington, GA, USA) and the transrectal ultrasound (TRUS)-guided, template-assisted technique. Patients were placed in the lithotomy position, and a biplanar transrectal ultrasound probe (Hitachi, Tokyo, Japan) was used for real-time guidance. A brachytherapy-type template grid (Computerized Medical System, St. Louis, MO, USA) was applied to the perineum to guide systematic sampling. A total of 12 systematic cores were obtained according to a modified template [18], and an additional 1–4 MRI-targeted cores were obtained per PI-RADS ≥3 lesion using cognitive-fusion registration. All biopsies were performed by three attending urologists (Yi Zhou, Zhien Zhou, and Weigang Yan), each with more than 10 years of experience in transperineal biopsy. Both LA and GA procedures were performed in the operating room.

For patients receiving local anesthesia, the institutional protocol comprised perineal skin infiltration with approximately 10 mL of 1% lidocaine (Otsuka Pharmaceutical (China) Co., Ltd., Tianjin, China), followed by bilateral periprostatic nerve block (PPNB) with 10 mL of 1% lidocaine injected around the prostate apex under ultrasound guidance [19]. No routine intravenous sedation was administered. For patients under general anesthesia, total intravenous anesthesia was delivered. Ventilation was performed via laryngeal mask airway (Aura-Gain, Ambu A/S, Ballerup, Denmark; manufactured by Ambu (Xiamen) Medical Devices Co., Ltd., Xiamen, China) or endotracheal intubation (Medtronic-Covidien, Fridley, MN, USA) for difficult airways. Induction utilized propofol (Diprivan, AstraZeneca, London, UK), fentanyl (Humanwell Pharmaceutical Co., Ltd., Yichang, China) and rocuronium (Esmeron, N.V. Organon, Oss, The Netherlands); midazolam (Liyuexi, Jiangsu Nhwa Pharmaceutical Co., Ltd., Xuzhou, China) was administered as required. Anesthesia was maintained with target-controlled propofol infusion and continuous remifentanil (Ruijie, Humanwell Pharmaceutical Co., Ltd., Yichang, China), and vasoactive medications were titrated according to hemodynamic status. For patients without contraindications to general anesthesia, the choice of anesthesia was made jointly by the patient and the attending urologist; patients with contraindications to general anesthesia underwent LA. Both modalities were available concurrently throughout the study period.

### 2.3. Histopathological Assessment

Biopsy specimens were processed according to standard institutional pathology protocols. Each core was individually labeled and submitted for histopathological examination. All specimens were reviewed by dedicated genitourinary pathologists. The presence and grade of prostate adenocarcinoma were reported according to the International Society of Urological Pathology (ISUP) 2014 grading system (Grade Groups 1–5) [20]. Clinically significant prostate cancer (csPCa) was defined as ISUP Grade Group ≥ 2 (Gleason score ≥ 3 + 4 = 7). High-grade prostate cancer was defined as ISUP Grade Group ≥ 3 (Gleason score ≥ 4 + 3 = 7). For each biopsy type (combined, systematic, and targeted), any Gleason score ≥ 3 + 3 = 6 was considered cancer-positive. The primary outcome was the csPCa detection rate on combined systematic and targeted biopsy (combined biopsy). Secondary outcomes included overall cancer detection rate, high-grade cancer detection rate, and detection rates by individual biopsy type.

For patients who subsequently underwent radical prostatectomy without neoadjuvant androgen-deprivation therapy, pathological concordance between biopsy and prostatectomy was assessed [21]. Concordance was assessed on the basis of ISUP Grade Group alone, rather than tumor location or volume. The interval between biopsy and radical prostatectomy was within 3 months in all patients. Endpoints included clinically significant upgrade (biopsy ISUP ≤ 1 to pathology ISUP ≥ 2), high-grade upgrade (biopsy ISUP ≤ 2 to pathology ISUP ≥ 3), any upgrade (biopsy ISUP < pathology ISUP), concordance (biopsy ISUP = pathology ISUP), any downgrade (biopsy ISUP > pathology ISUP), and clinically significant downgrade (biopsy ISUP ≥ 2 to pathology ISUP = 1).

### 2.4. Statistical Analysis

Continuous variables were expressed as mean ± standard deviation (SD) or median (interquartile range), and compared between the LA and GA groups using Student’s independent-samples *t*-test or the Mann–Whitney U test, as appropriate. Categorical variables were expressed as frequencies and percentages and compared using the chi-squared test or Fisher’s exact test.

To account for baseline differences between groups, 1:1 propensity score matching (PSM) was performed [22]. A logistic regression model estimated the probability of receiving GA based on age, PSA, PI-RADS score, and prostate volume, which are established predictors of csPCa and could plausibly influence the choice of anesthesia [17,23]. For patients with multiple lesions, the highest PI-RADS category was used for matching. Nearest-neighbor matching without replacement was applied with a caliper of 0.2 standard deviations of the logit of the propensity score. Primary analyses were conducted in the PSM cohort. The target estimand was the average treatment effect on the treated (ATT); the matched cohort therefore represents biopsy-naive men eligible for LA.

Pre-specified subgroup analyses for csPCa detection were performed, stratifying by age, prostate volume, PSA level, PSAD, and PI-RADS score. Univariable and multivariable logistic regression identified independent predictors of csPCa. PSA and PSAD were not entered simultaneously (PSAD = PSA/prostate volume). The results are presented as odds ratios (ORs) or adjusted odds ratios (aORs) with 95% confidence intervals (CIs). All statistical tests were two-sided (*p* < 0.05 considered significant). Analyses were performed using open-source Python (version 3.12; Python Software Foundation, Wilmington, DE, USA) with the SciPy (1.15.2), pandas (2.2.3), statsmodels (0.14.6), and scikit-learn (1.9.0) libraries [24]. A post hoc power analysis was performed for the primary outcome using the observed event rate in the LA group, a two-sided significance level of 0.05, and a matched sample size of 220 per group.

## 3. Results

**Figure 1:**
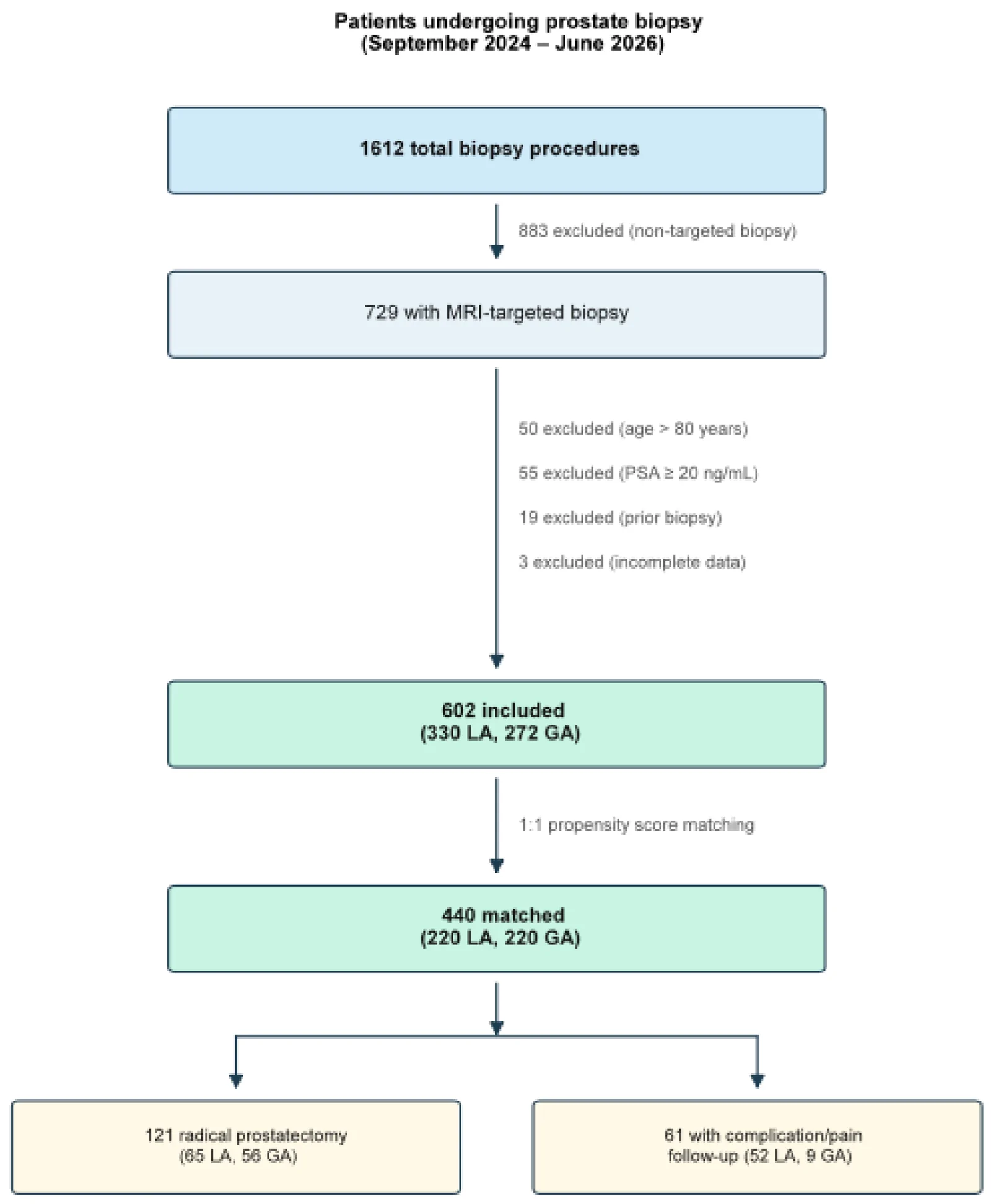
STROBE flow diagram of patient screening and inclusion.

From September 2024 to June 2026, 1612 patients were screened; after excluding 883 with non-targeted biopsy, 50 aged > 80 years, 55 with PSA ≥ 20 ng/mL, 19 with prior biopsy, and 3 with incomplete data, 602 biopsy-naive men were included (Figure 1). Of these, 330 (54.8%) received LA and 272 (45.2%) received GA. Before PSM, the LA group was older than the GA group (mean age 67.5 ± 6.8 vs. 65.7 ± 7.5 years, *p* = 0.002). PSA, PSAD, prostate volume, and PI-RADS did not differ significantly between groups (Table 1). The median total number of biopsy cores was 14 in both groups; however, a higher proportion of GA patients had ≥15 cores (9.5% vs. 4.1%, *p* = 0.036) and ≥2 MRI lesions (7.7% vs. 3.2%, *p* = 0.057), while cores per target did not differ (≥3 cores: 2.3% vs. 0.9%, *p* = 0.449) (Table 1). After 1:1 PSM, 220 matched pairs (440 patients) were retained. One hundred and ten LA and 52 GA patients were excluded during matching (162 of 602). In the matched cohort, before matching, the absolute SMDs were 0.248 for age, 0.023 for PSA, 0.072 for prostate volume, and 0.040 for PI-RADS. After matching, covariate balance was achieved, with absolute standardized mean differences below 0.15 for all variables (age 0.029, PSA 0.129, prostate volume 0.017, PI-RADS 0.030; Love plot in Supplementary Materials). Abnormal DRE was observed in 58/220 (26.4%) of the LA group and 46/220 (20.9%) of the GA group (*p* = 0.217). LA and GA were used concurrently throughout the study period, with the quarterly proportion of LA procedures ranging from 45% to 77%.

### 3.1. Cancer Detection Rates

**Figure 2:**
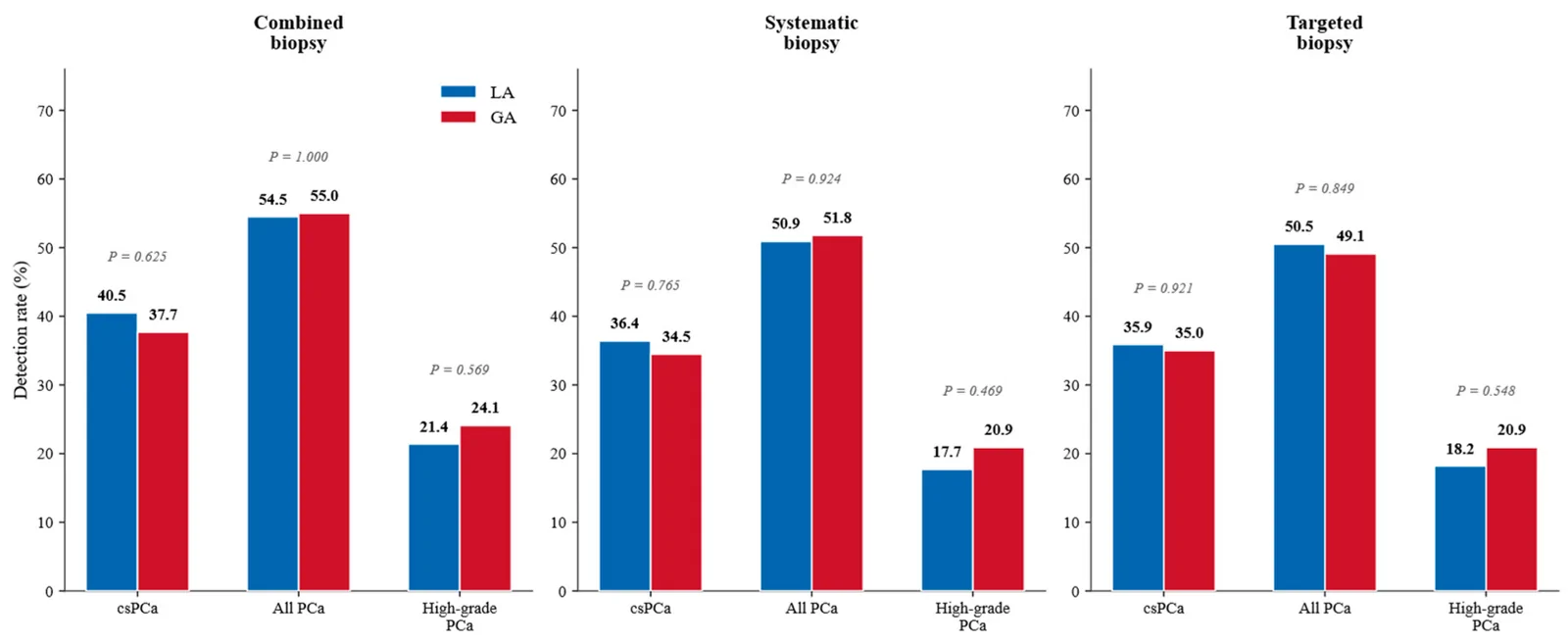
Cancer detection rates of combined, systematic, and targeted biopsy in the propensity score-matched cohort (220 matched pairs). No significant difference was observed between the local anesthesia and general anesthesia groups across any biopsy modality or outcome (all *p* > 0.05). LA = local anesthesia; GA = general anesthesia; csPCa = clinically significant prostate cancer (ISUP Grade Group ≥ 2); PCa = prostate cancer; High-grade PCa = ISUP Grade Group ≥ 3.

In the propensity score-matched cohort, the csPCa detection rate on combined biopsy was 40.5% (89/220) in the LA group and 37.7% (83/220) in the GA group (*p* = 0.625). At the observed baseline rate of 40.5% and 220 patients per group, this cohort had 80% power to detect an absolute difference in csPCa detection of approximately 13.3 percentage points (two-sided α = 0.05). The overall cancer detection rate was 54.5% (120/220) vs. 55.0% (121/220) (*p* = 1.000), and the high-grade cancer detection rate was 21.4% (47/220) vs. 24.1% (53/220) (*p* = 0.569). None of these comparisons reached statistical significance (Figure 2). The absolute risk difference (GA minus LA) for csPCa detection was −2.7 percentage points (95% CI −11.8 to 6.4) and the relative risk was 0.93 (95% CI 0.74–1.18); for overall cancer detection, the absolute risk difference was 0.5 percentage points (95% CI −8.8 to 9.8) and the relative risk was 1.01 (95% CI 0.85–1.19); for high-grade cancer detection, the absolute risk difference was 2.7 percentage points (95% CI −5.1 to 10.6) and the relative risk was 1.13 (95% CI 0.80–1.59). A paired sensitivity analysis using McNemar’s test on the 220 matched pairs yielded consistent results for csPCa detection (*p* = 0.627).

Similarly, no significant differences were observed for systematic biopsy or targeted biopsy individually. Systematic biopsy csPCa detection rates were 36.4% (80/220) vs. 34.5% (76/220) (*p* = 0.765), and overall cancer detection rates were 50.9% (112/220) vs. 51.8% (114/220) (*p* = 0.924). Targeted biopsy csPCa detection rates were 35.9% (79/220) vs. 35.0% (77/220) (*p* = 0.921), and overall cancer detection rates were 50.5% (111/220) vs. 49.1% (108/220) (*p* = 0.849). The complete detection rates for all biopsy types are presented in Figure 2. Cancer detection rates in the full unmatched cohort are reported in Supplementary Table S1.

### 3.2. Subgroup Analyses

Pre-specified exploratory subgroup analyses stratified by age (<60, ≥60 to <70, ≥70 years), prostate volume (<50 vs. ≥50 mL), PSA level (<4, ≥4 to <10, ≥10 to <20 ng/mL), PSAD (<0.15, ≥0.15 to <0.30, ≥0.30), and PI-RADS score (3, 4, 5) did not show significant differences in combined csPCa detection between the LA and GA groups in individual strata (Table 2). The nominally larger differences observed in the PSA ≥ 10 to <20 ng/mL stratum (LA 65.5% vs. GA 47.8%, *p* = 0.076) and the PI-RADS 3 stratum (LA 0% vs. GA 15.2%, *p* = 0.053) did not reach statistical significance and should be interpreted with caution given the small subgroup sample sizes. Given the number of subgroup comparisons and the small sample sizes in some strata (particularly PI-RADS 3), these analyses are susceptible to both false-positive and false-negative findings. Subgroup analyses in the full unmatched cohort are shown in Supplementary Table S2.

### 3.3. Logistic Regression Analyses

**Figure 3:**
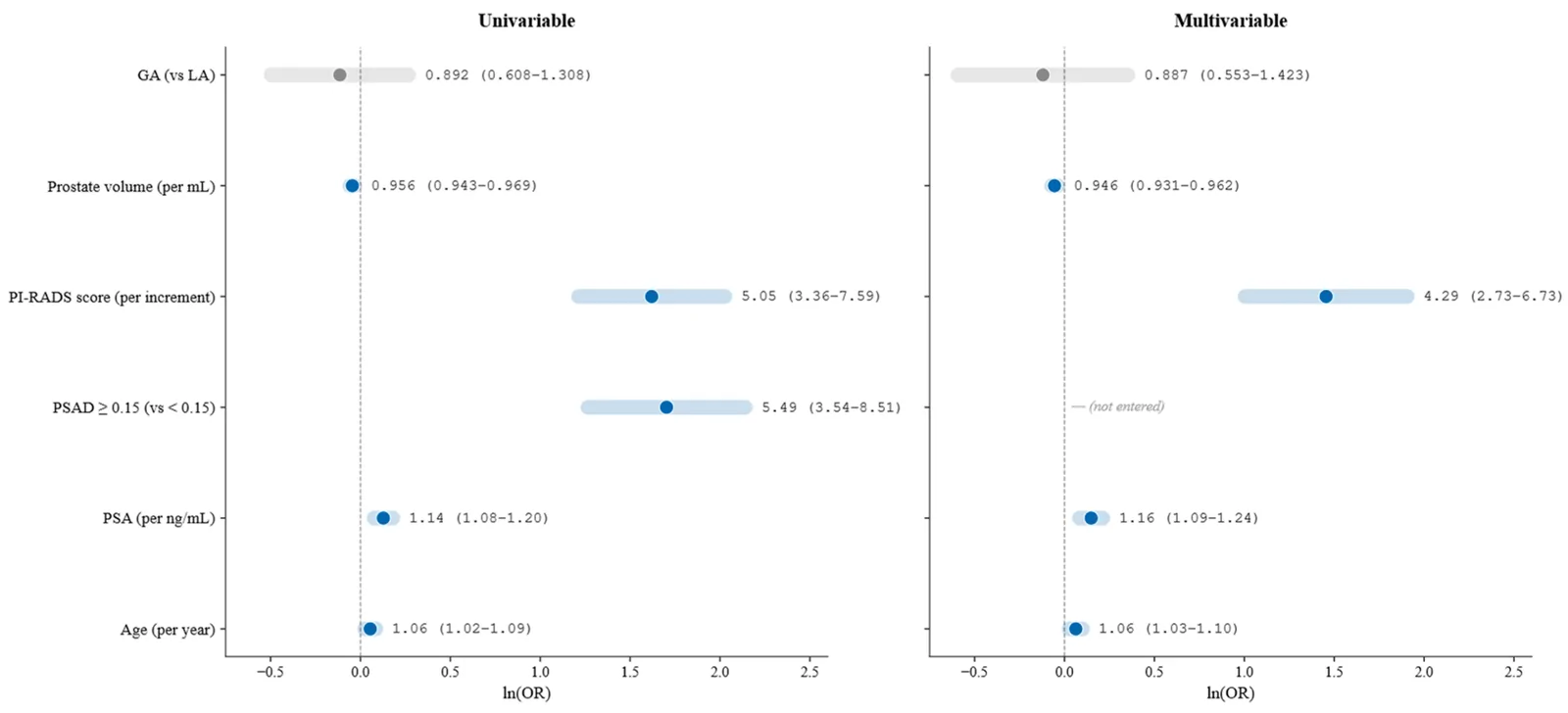
Forest plot of univariable and multivariable logistic regression for clinically significant prostate cancer detection in the propensity score-matched cohort. Anesthesia type (GA vs. LA) was not an independent predictor of csPCa (adjusted OR 0.887, 95% CI 0.553–1.423, *p* = 0.620), while PI-RADS score, PSA, age, and prostate volume were significant independent predictors. PSAD was excluded from the multivariable model due to collinearity (PSAD = PSA/prostate volume).

On univariable logistic regression in the matched cohort, age (OR 1.06 per year, 95% CI 1.02–1.09, *p* < 0.001), PSA (OR 1.14 per ng/mL, 95% CI 1.08–1.20, *p* < 0.001), PSAD ≥ 0.15 ng/mL^2^ (OR 5.40, 95% CI 3.48–8.37, *p* < 0.001), PI-RADS score (OR 5.09 per increment, 95% CI 3.39–7.66, *p* < 0.001), and prostate volume (OR 0.96 per mL, 95% CI 0.94–0.97, *p* < 0.001) were all significantly associated with combined csPCa detection. Anesthesia type was not significantly associated with csPCa detection (GA vs. LA: OR 0.892, 95% CI 0.608–1.308, *p* = 0.558) (Figure 3).

On multivariable logistic regression adjusting for age, PSA, PI-RADS score, and prostate volume, the independent predictors of combined csPCa were age (aOR 1.06 per year, 95% CI 1.02–1.10, *p* = 0.001), PSA (aOR 1.16 per ng/mL, 95% CI 1.09–1.24, *p* < 0.001), PI-RADS score (aOR 4.32 per increment, 95% CI 2.75–6.78, *p* < 0.001), and prostate volume (aOR 0.95 per mL, 95% CI 0.93–0.96, *p* < 0.001). Anesthesia type was not an independent predictor of csPCa detection (GA vs. LA: aOR 0.887, 95% CI 0.553–1.423, *p* = 0.620). The results of univariable and multivariable analyses in the full unmatched cohort were consistent with those in the matched cohort and are presented in Supplementary Table S3.

### 3.4. Pathology Concordance

Of 440 patients in the PSM cohort, 121 underwent radical prostatectomy without neoadjuvant androgen-deprivation therapy and were eligible for pathology concordance analysis (LA: 65, GA: 56). Concordance rates between biopsy ISUP and prostatectomy ISUP did not differ significantly between the LA and GA groups across all endpoints. On combined biopsy, ISUP concordance was 56.9% and 51.8% respectively (*p* = 0.588). Among patients with biopsy ISUP ≤ 1, clinically significant upgrade rates were 38.5% (5/13) in the LA group and 61.5% (8/13) in the GA group (*p* = 0.434), and clinically significant downgrade rates (among biopsy ISUP ≥ 2) were 5.8% (3/52) vs. 4.7% (2/43) (*p* = 1.000). All between-group comparisons for pathological concordance on combined biopsy are presented in Table 3. Concordance analyses for systematic and targeted biopsy are provided in Supplementary Tables S5 and S6. Given the small number of prostatectomy patients, this subset had 80% power only to detect an absolute difference of approximately 23.8 percentage points in Grade Group concordance, so these concordance findings are exploratory and should be interpreted with caution.

## 4. Discussion

In this single-center retrospective study of 602 biopsy-naive men undergoing cognitive-fusion transperineal prostate biopsy, we found that anesthesia type—local anesthesia versus general anesthesia—was not independently associated with csPCa detection, overall cancer detection, or high-grade cancer detection. This null association persisted across all three biopsy modalities (combined, systematic, and targeted) and remained robust after propensity score matching on age, PSA, PI-RADS score, and prostate volume. On multivariable logistic regression, the adjusted odds ratio for GA versus LA was 0.887 (95% CI 0.553–1.423, *p* = 0.620), indicating no statistically significant association between anesthesia type and csPCa detection. The wide 95% confidence interval (0.553–1.423) remains compatible with clinically relevant differences in either direction and therefore does not establish formal equivalence between the two approaches.

Our finding that anesthesia type does not independently influence csPCa detection is consistent with the majority of prior comparative studies; however, each of them differs from the present work in terms of important design features. Hogan et al., in a pilot study of 80 patients, reported comparable overall and csPCa detection between LA and GA; notably, the LA group had a higher median PI-RADS score at baseline (4 vs. 3, *p* = 0.033), which may have biased the comparison in favor of LA [7]. Lv et al. conducted the only RCT to date (n = 216) and found no significant difference in cancer detection between LA and GA TP biopsy [12]; however, their cohort was notably high-risk (mean PSA ≈ 22 ng/mL, 75–83% DRE-positive), and pre-biopsy MRI was not required for all participants, limiting generalizability to contemporary MRI-targeted practice. Zaurito et al. (n = 1021) also found no difference in targeted biopsy csPCa detection [15]. Vasconcelos Ordones et al. reported higher csPCa detection with in-office LA cognitive-fusion biopsy than with GA software-fusion biopsy in a multicenter comparison of 1272 patients, but this result was likely confounded by between-center differences in operator experience—the LA cohort was treated by two high-volume specialists (>1000 cases each), while the GA cohort included trainees [13]. Among prior studies, only Kim et al. reported a significant advantage for GA (propofol sedation) over LA (intrarectal lidocaine gel) in a retrospective cohort of 2119 men (34.0% vs. 29.2%, *p* = 0.024 after adjustment) [11]. This divergent finding is likely attributable to key methodological differences: Kim et al. employed the transrectal rather than transperineal route, and the LA regimen was limited to intrarectal gel without periprostatic or pudendal nerve block—a less comprehensive analgesic strategy than current TP protocols; moreover, the sedation and LA cohorts were temporally separated, potentially introducing era effects. By employing a uniform single-center cognitive-fusion TP biopsy protocol and propensity score matching, our study isolates the anesthesia variable independently of differences in biopsy route, fusion method, LA protocol, and operator experience. Taken together, the available evidence suggests that in a TP setting with adequate LA protocols, anesthesia type does not meaningfully affect diagnostic yield.

The theoretical mechanisms by which GA has been proposed to facilitate cancer detection find limited empirical support. Hogan et al. observed no difference in the number of biopsy cores obtained under GA versus LA [7], and Chung and Hong noted that the perception of LA-TP as procedurally inadequate is largely attributable to its historical association with brachytherapy-grid techniques rather than to intrinsic limitations of the LA approach [25]. The present finding that these theoretical mechanisms do not translate into a clinically meaningful detection advantage for GA is therefore consistent with the broader evidence base.

From a mechanistic standpoint, the absence of an anesthesia-dependent difference in cancer detection is biologically plausible. The theoretical argument favoring GA—that elimination of patient movement improves needle placement accuracy—may be less relevant in the transperineal setting, where the template grid and ultrasound guidance provide inherent spatial stabilization. This is consistent with the recent TRANSLATE RCT [26], which demonstrated that LA-TP biopsy yields superior csPCa detection compared with the TRUS approach. Furthermore, cognitive fusion, as employed in our cohort, has been shown to achieve comparable csPCa detection to software-assisted fusion [27], and relies on operator interpretation of pre-biopsy MRI rather than real-time software registration [28]; any incremental beneficial effect of GA on targeting precision may be smaller than the dominant effect of operator experience and lesion conspicuity. The recent observation that higher intra-procedural pain during LA was associated with lower csPCa detection on interaction testing suggests that optimizing pain control within LA protocols—rather than abandoning LA in favor of GA—represents the more appropriate clinical strategy [15].

Accurate pathological concordance between biopsy and prostatectomy is essential for reliable risk stratification and treatment decision-making [29]. Anesthesia type did not significantly influence the agreement between biopsy ISUP grade and radical prostatectomy specimen ISUP grade in the subset of 121 patients with evaluable prostatectomy pathology within the PSM cohort. Across combined, systematic, and targeted biopsy, rates of clinically significant upgrade, high-grade upgrade, concordance, and downgrade were comparable between the LA and GA groups (all *p* > 0.05; Supplementary Tables S5 and S6). To our knowledge, this represents the first evaluation of the impact of anesthesia type on pathological concordance between biopsy and radical prostatectomy; no prior studies comparing LA and GA for prostate biopsy have reported concordance, upgrade, or downgrade rates. These findings provide the first evidence that the choice of anesthesia does not materially affect the reliability of biopsy-based risk stratification, although the limited sample size precludes definitive conclusions. Future prospective studies incorporating larger prostatectomy-confirmed cohorts are warranted to validate these observations.

These findings support the continued use of LA-TP biopsy, although the confidence intervals do not establish formal equivalence between LA and GA. The elimination of general anesthesia from the biopsy pathway has the potential to reduce procedural costs [30], free operating room capacity, and avoid the cardiovascular and respiratory risks associated with GA, particularly in an aging population with significant comorbidity burdens [8,14]. However, GA may still be appropriately reserved for specific clinical scenarios—such as patients with severe anxiety, inability to tolerate the lithotomy position while awake, or very large prostate glands requiring extended sampling [31]—rather than being considered the default standard.

Several limitations of this study warrant acknowledgment. First, its retrospective, non-randomized design introduces the potential for selection bias and confounding by indication; patients receiving GA may have differed from those receiving LA in unmeasured characteristics such as pain tolerance, baseline anxiety, or anatomical complexity. Index-lesion size and location, ASA class, and operator-specific case distribution were not available for analysis and represent additional potential sources of residual confounding. Missing data for prostate volume and PSA density were handled by excluding those patients during propensity score matching. Although PSM balanced the groups on measured covariates, residual confounding cannot be excluded. Because this was a retrospective study that consecutively enrolled eligible patients, no a priori sample size calculation was performed, and the null findings should be interpreted accordingly. Second, this was a single-center study conducted at a high-volume academic institution with experienced operators and dedicated genitourinary radiologists and pathologists; the generalizability of these findings to lower-volume community settings requires independent validation. Third, all biopsies employed cognitive fusion rather than software-assisted fusion; whether the relationship between anesthesia type and cancer detection differs under software-fusion conditions remains an open question. Moreover, all procedures used a template grid, which provides spatial stabilization that may reduce the impact of patient movement; the findings may therefore not generalize to freehand transperineal biopsy, software-assisted fusion, or robotic platforms, where patient immobility may contribute differently to targeting accuracy. Fourth, the LA protocol employed at our institution did not include routine pudendal nerve block; whether more comprehensive LA techniques might further narrow any residual differences in pain control or diagnostic performance warrants investigation, although available evidence suggests that periprostatic nerve block with perineal infiltration provides analgesia comparable to more comprehensive techniques [32], so this omission is unlikely to have materially biased the comparison against LA. Finally, follow-up data were limited: complication and pain data were available for only 61 of 440 matched patients (52 LA, 9 GA). No LA procedure was aborted or converted to general anesthesia, and no rescue sedation was required. Among these patients (Supplementary Table S7), complication rates including hematuria and hematospermia were similar between groups, with no severe Clavien-Dindo ≥3 complications or sepsis; VAS pain scores were numerically lower in the GA group than in the LA group (1.78 vs. 2.88, *p* = 0.161), although this difference was not statistically significant; VAS was self-reported at the 2-week outpatient or telephone follow-up, and given that follow-up was incomplete and no long-term oncologic outcomes were assessed, these data do not support a claim of oncologic safety.

## 5. Conclusions

In conclusion, among biopsy-naive men undergoing cognitive-fusion transperineal prostate biopsy, anesthesia type was not independently associated with the detection of csPCa or high-grade PCa. These findings support the feasibility of LA transperineal biopsy; although the confidence intervals do not establish formal equivalence between the two approaches, anesthesia modality may be selected based on patient preference, anatomic considerations, and institutional resources rather than concern for differential diagnostic performance.

## Figures and Tables

**Table 1:** Baseline characteristics before and after PSM.

Variable	LA (n = 330)	GA (n = 272)	*p* (Before)	LA (n = 220)	GA (n = 220)	*p* (After)
Age (years)	67.45 ± 6.82	65.68 ± 7.45	0.002	66.25 ± 6.69	66.45 ± 6.63	0.758
PSA (ng/mL)	7.32 (5.44–10.30)	7.31 (5.23–10.05)	0.669	7.49 (5.33–10.23)	6.79 (4.99–9.39)	0.131
PSAD (ng/mL^2^)	0.160 (0.100–0.245)	0.160 (0.106–0.222)	0.870	0.162 (0.102–0.259)	0.156 (0.105–0.213)	0.298
Prostate volume (mL)	45 (34–60)	45 (32–60)	0.345	43.5 (32–60)	43 (31–58)	0.635
Total biopsy cores ≥ 15, n (%)	13 (3.9%)	27 (9.9%)	0.005	9 (4.1%)	21 (9.5%)	0.036
MRI lesions ≥ 2, n (%)	10 (3.0%)	22 (8.1%)	0.010	7 (3.2%)	17 (7.7%)	0.057
Cores per target ≥ 3, n (%)	3 (0.9%)	6 (2.2%)	0.312	2 (0.9%)	5 (2.3%)	0.449
PI-RADS 3, n (%)	38 (11.5%)	42 (15.4%)	0.279	31 (14.1%)	34 (15.5%)	0.922
PI-RADS 4, n (%)	197 (59.7%)	148 (54.4%)		138 (62.7%)	136 (61.8%)	
PI-RADS 5, n (%)	95 (28.8%)	82 (30.1%)		51 (23.2%)	50 (22.7%)	

Data are mean ± SD, median (interquartile range), or n (%). Non-normally distributed continuous variables (PSA, PSAD, and prostate volume) are presented as median (interquartile range). *p* values: *t*-test or Mann–Whitney U test for continuous variables, chi-squared test or Fisher’s exact test for categorical variables. LA = local anesthesia; GA = general anesthesia; PSA = prostate-specific antigen; PSAD = PSA density; PI-RADS = Prostate Imaging Reporting and Data System; PSM = propensity score matching.

**Table 2:** Subgroup analysis of csPCa detection in the PSM cohort of combined biopsy.

Subgroup	Stratum	LA, n/N (%)	GA, n/N (%)	*p* Value
Age	<60	13/34 (38.2%)	6/29 (20.7%)	0.172
	≥60 to <70	42/113 (37.2%)	43/113 (38.1%)	1.000
	≥70	34/73 (46.6%)	34/78 (43.6%)	0.745
PSA (ng/mL)	<4	5/24 (20.8%)	7/32 (21.9%)	1.000
	≥4 to <10	46/138 (33.3%)	54/142 (38.0%)	0.455
	≥10 to <20	38/58 (65.5%)	22/46 (47.8%)	0.076
Prostate volume (mL)	≥50	16/90 (17.8%)	17/87 (19.5%)	0.848
	<50	73/130 (56.2%)	66/133 (49.6%)	0.324
PSAD (ng/mL^2^)	<0.15	19/95 (20.0%)	18/103 (17.5%)	0.717
	≥0.15 to <0.30	39/86 (45.3%)	41/87 (47.1%)	0.879
	≥0.30	31/39 (79.5%)	24/30 (80.0%)	1.000
PI-RADS	3	0/31 (0.0%)	5/33 (15.2%)	0.053
	4	52/138 (37.7%)	43/136 (31.6%)	0.312
	5	37/51 (72.5%)	35/50 (70.0%)	0.828

csPCa = clinically significant prostate cancer (ISUP Grade Group ≥ 2). *p* values according to Fisher’s exact test. LA = local anesthesia; GA = general anesthesia; PSA = prostate-specific antigen; PSAD = PSA density; PI-RADS = Prostate Imaging Reporting and Data System. Age and PSAD strata are defined as left-closed, right-open intervals.

**Table 3:** Pathological concordance between combined biopsy and radical prostatectomy specimen in the PSM cohort.

Endpoint	LA, n/N (%)	GA, n/N (%)	*p* Value
csPCa upgrade (ISUP ≤ 1 → ≥2)	5/13 (38.5%)	8/13 (61.5%)	0.434
High-grade upgrade (ISUP ≤ 2 → ≥3)	5/38 (13.2%)	5/29 (17.2%)	0.736
csPCa downgrade (ISUP ≥ 2 → ≤1)	3/52 (5.8%)	2/43 (4.7%)	1.000
Any upgrade (biopsy < pathology)	14/65 (21.5%)	12/56 (21.4%)	1.000
ISUP concordance (biopsy = pathology)	37/65 (56.9%)	29/56 (51.8%)	0.588
Any downgrade (biopsy > pathology)	14/65 (21.5%)	15/56 (26.8%)	0.528

csPCa = clinically significant prostate cancer (ISUP Grade Group ≥ 2); LA = local anesthesia; GA = general anesthesia; PSM = propensity score matching; ISUP = International Society of Urological Pathology.

## Data Availability

The data used to support the findings of this study are available from the corresponding author on request.

## Supplementary Materials

The following supporting information can be downloaded at https://www.mdpi.com/article/10.3390/cancers18172820/s1, Supplementary Table S1. Cancer detection rates in all patients. Supplementary Table S2. Subgroup analysis of csPCa detection in all patient. Supplementary Table S3. Logistic regression for fusion csPCa in all patients. Supplementary Table S4. Baseline characteristics of the prostatectomy subset (n = 121). Supplementary Table S5. Pathology concordance in PSM cohort for systematic biopsy. Supplementary Table S6. Pathology concordance in PSM cohort for targeted biopsy. Supplementary Table S7. Procedure-related complications in the PSM cohort. Supplementary Figure S1. Love plot of absolute standardized mean differences (SMD) before and after propensity score matching. The dashed vertical line indicates the 0.1 threshold.

## Abbreviations

The following abbreviations are used in this manuscript:

csPCa	Clinically Significant Prostate Cancer
PCa	Prostate Cancer
LA	Local Anesthesia
GA	General Anesthesia
TP	Transperineal
TRUS	Transrectal Ultrasound
MRI	Magnetic Resonance Imaging
mpMRI	Multiparametric MRI
PSA	Prostate-Specific Antigen
PSAD	PSA Density
PI-RADS	Prostate Imaging Reporting and Data System
ISUP	International Society of Urological Pathology
GG	Grade Group
PPNB	Periprostatic Nerve Block
TIVA	Total Intravenous Anesthesia
LMA	Laryngeal Mask Airway
ETT	Endotracheal Tube
PSM	Propensity Score Matching
OR	Odds Ratio
aOR	Adjusted Odds Ratio
CI	Confidence Interval
SD	Standard Deviation
DRE	Digital Rectal Examination
STROBE	Strengthening the Reporting of Observational Studies in Epidemiology
EAU	European Association of Urology
RCT	Randomized Controlled Trial
NICE	National Institute for Health and Care Excellence
AUA	American Urological Association
VAS	Visual Analog Scale
