# Supplementary material for: Effect of Anesthesia Type on Clinically Significant Prostate Cancer Detection in Transperineal MRI-Targeted Biopsy
Source: Cancers (Basel). 2026 Sep 1;18(17):2820. doi: 10.3390/cancers18172820 (PMC13564555; doi:10.3390/cancers18172820)

Effect of Anesthesia Type on Clinically Significant Prostate Cancer Detection in Transperineal MRI-Targeted Biopsy

Supplementary Materials

Supplementary Table S1. Cancer detection rates in all patients

Modality	Outcome	LA (n=330)	GA (n=272)	P value
Fusion biopsy	csPCa (ISUP >=2)	130/330 (39.4%)	97/272 (35.7%)	0.354
Fusion biopsy	All PCa (ISUP >=1)	181/330 (54.8%)	142/272 (52.2%)	0.565
Fusion biopsy	High-grade PCa (ISUP >=3)	71/330 (21.5%)	64/272 (23.5%)	0.558
Systematic biopsy	csPCa (ISUP >=2)	113/330 (34.2%)	87/272 (32.0%)	0.602
Systematic biopsy	All PCa (ISUP >=1)	164/330 (49.7%)	134/272 (49.3%)	0.935
Systematic biopsy	High-grade PCa (ISUP >=3)	57/330 (17.3%)	56/272 (20.6%)	0.345
Targeted biopsy	csPCa (ISUP >=2)	113/330 (34.2%)	86/272 (31.6%)	0.542
Targeted biopsy	All PCa (ISUP >=1)	159/330 (48.2%)	125/272 (46.0%)	0.623
Targeted biopsy	High-grade PCa (ISUP >=3)	60/330 (18.2%)	53/272 (19.5%)	0.753

Supplementary Table S2. Subgroup analysis of csPCa detection in all patient

Subgroup	Stratum	LA, n/N (%)	GA, n/N (%)	P value
Age	<60	14/40 (35.0%)	9/47 (19.1%)	0.143
	60-70	57/149 (38.3%)	48/131 (36.6%)	0.806
	>70	59/141 (41.8%)	40/94 (42.6%)	1.000
Prostate volume	<50 mL	97/180 (53.9%)	73/155 (47.1%)	0.229

	>=50 mL	29/142 (20.4%)	20/112 (17.9%)	0.634
PSA	<4	6/34 (17.6%)	8/36 (22.2%)	0.768
	4-10	67/208 (32.2%)	61/167 (36.5%)	0.384
	10-20	57/88 (64.8%)	28/69 (40.6%)	0.004
PSAD	<0.15	24/144 (16.7%)	20/119 (16.8%)	1.000
	0.15-0.30	60/127 (47.2%)	47/110 (42.7%)	0.515
	>0.30	42/51 (82.4%)	26/38 (68.4%)	0.139
PI-RADS	3	2/38 (5.3%)	5/41 (12.2%)	0.434
	4	72/197 (36.5%)	47/148 (31.8%)	0.363
	5	49/71 (69.0%)	37/53 (69.8%)	1.000

Supplementary Table S3. Logistic regression for fusion csPCa in all patients

Variable	OR (95% CI)	P (Univariable)	aOR (95% CI)	P (Multivariable)
Age (per year)	1.046 (1.019-1.073)	0.001	1.055 (1.023-1.089)	0.001
PSA (per ng/mL)	1.131 (1.079-1.185)	0.000	1.173 (1.106-1.244)	0.000
PSAD >=0.15	5.807 (3.877-8.698)	0.000	— (not entered)	—
PI-RADS score	4.894 (3.380-7.086)	0.000	4.277 (2.832-6.461)	0.000
Prostate volume (per mL)	0.956 (0.945-0.968)	0.000	0.947 (0.933-0.960)	0.000
GA (vs LA)	0.845 (0.595-1.202)	0.349	0.888 (0.576-1.370)	0.592

Supplementary Table S4. Baseline characteristics of the prostatectomy subset (n = 121).

Variable	LA (n=65)	GA (n=56)
Age (years), median (IQR)	67 (62–72)	68 (64–71)
PSA (ng/mL), median (IQR)	7.3 (5.6–11.2)	7.9 (5.2–11.1)
PSAD (ng/mL^2), median (IQR)	0.195 (0.134–0.280)	0.195 (0.153–0.308)
Prostate volume (mL), median (IQR)	40 (32–50)	40 (30–46)
PI-RADS 3, n (%)	2 (3.1%)	1 (1.8%)
PI-RADS 4, n (%)	41 (63.1%)	34 (60.7%)
PI-RADS 5, n (%)	22 (33.8%)	21 (37.5%)

Values are median (interquartile range) or n (%). P values: Mann-Whitney U test for continuous variables and Fisher exact test for PI-RADS. All between-group P values were > 0.05.

Supplementary Table S5. Pathology concordance in PSM cohort for systematic biopsy

Endpoint	LA, n/N (%)	GA, n/N (%)	P value
csPCa upgrade (ISUP ≤1 → ≥2)	11/19 (57.9%) [33.5–79.7]	10/15 (66.7%) [38.4–88.2]	0.728
High-grade upgrade (ISUP ≤2 → ≥3)	9/44 (20.5%) [9.8–35.3]	8/32 (25.0%) [11.5–43.4]	0.782
csPCa downgrade (ISUP ≥2 → ≤1)	3/46 (6.5%) [1.4–17.9]	2/41 (4.9%) [0.6–16.5]	1.000
Any upgrade (biopsy < pathology)	24/65 (36.9%)	16/56 (28.6%)	0.342
ISUP concordance (biopsy = pathology)	29/65 (44.6%)	27/56 (48.2%)	0.718
Any downgrade (biopsy > pathology)	12/65 (18.5%)	13/56 (23.2%)	0.653

Supplementary Table S6. Pathology concordance in PSM cohort for targeted biopsy

Endpoint	LA, n/N (%)	GA, n/N (%)	P value
csPCa upgrade (ISUP ≤1 → ≥2)	7/17 (41.2%) [18.4–67.1]	13/18 (72.2%) [46.5–90.3]	0.092
High-grade upgrade (ISUP ≤2 → ≥3)	8/43 (18.6%) [8.4–33.4]	7/34 (20.6%) [8.7–37.9]	1.000
csPCa downgrade (ISUP ≥2 → ≤1)	1/48 (2.1%) [0.1–11.1]	2/38 (5.3%) [0.6–17.7]	0.581
Any upgrade (biopsy < pathology)	22/65 (33.8%)	21/56 (37.5%)	0.707
ISUP concordance (biopsy = pathology)	34/65 (52.3%)	25/56 (44.6%)	0.467
Any downgrade (biopsy > pathology)	9/65 (13.8%)	10/56 (17.9%)	0.620

Supplementary Table S7. Procedure-related complications in the PSM cohort

Complication	LA	GA	P value
Hematospermia	8/52 (15.4%)	2/9 (22.2%)	0.633
Hematuria	44/52 (84.6%)	8/9 (88.9%)	1.000
Urinary retention	1/52 (1.9%)	0/9 (0.0%)	1.000
Urinary frequency/urgency	4/52 (7.7%)	0/9 (0.0%)	1.000
Perineal hematoma	4/52 (7.7%)	1/9 (11.1%)	0.563

Rectal bleeding	1/52 (1.9%)	0/9 (0.0%)	1.000
Pain VAS (mean +/- SD)	2.88 ± 2.11	1.78 ± 2.44	0.161

Complications were assessed by questionnaire at the 2-week outpatient visit or by telephone follow-up. Definitions: hematuria, macroscopic hematuria; hematospermia, macroscopic hematospermia; urinary retention, inability to void spontaneously requiring catheterization; perineal hematoma, patient-perceived subcutaneous swelling of the perineal region; rectal bleeding, anal bleeding or blood in the stool, excluding hemorrhoids and gastrointestinal bleeding. VAS = visual analog scale; pain was self-rated by the patient at the 2-week follow-up.

Supplementary Figure S1. Love plot of absolute standardized mean differences (SMD) before and after propensity score matching. The dashed vertical line indicates the 0.1 threshold.

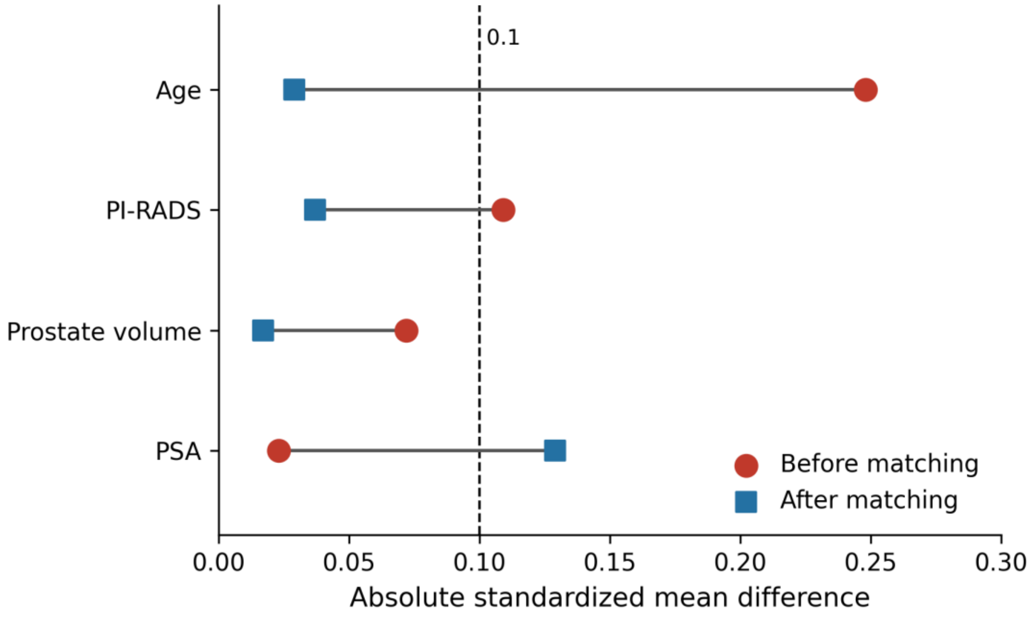

Supplement: Supplementary file 1 [file cancers-18-02820-s001.zip › cancers-4497615-supplementary.pdf]
